# Risk Factors of Central Line-Associated Bloodstream Infection (CLABSI): A Prospective Study From a Paediatric Intensive Care Unit in South India

**DOI:** 10.7759/cureus.43349

**Published:** 2023-08-11

**Authors:** Ravina Sellamuthu, Sajitha Nair, Jayakumar Chandrasekar, Sajith Kesavan, Vishnu Shivam

**Affiliations:** 1 Pediatrics, Amrita Institute of Medical Sciences and Research Centre, Kochi, IND; 2 Research and Development, Vedanadhi, Salem, IND

**Keywords:** risk factors, picu, central venous catheter, healthcare associated infection, clabsi, central line, bacteremia

## Abstract

Background

Central line-associated bloodstream infection (CLABSI) is the most common hospital-acquired infection. However, studies evaluating the factors associated with the risk of CLABSI in pediatric intensive care units (PICU) were limited in India.

Objective

The objective of our study is to evaluate the association of factors and the etiology causing CLABSI.

Study design

This is a hospital-based single-center prospective study conducted in the pediatric intensive care unit (PICU) of our tertiary care hospital spanning one year.

Participants

Children aged between two months to 15 years admitted in the PICU for more than 48 hours with central venous catheterization were included. Pearson's chi-squared test with Yates' continuity correction and logistic regression with odds ratio were calculated by R statistical software (R Foundation for Statistical Computing, Vienna, Austria) and a p-value less than 0.05 was considered statistically significant.

Results

Our analysis showed that factors such as young age (2-12 months), high pediatric risk of mortality (PRISM III) score (> 15), leukocytosis, neutrophilia, anemia, change of central venous catheter, duration of catheterization (>7 days), exposure to blood products, use of steroids, inotropes, and prophylactic antibiotics were significantly associated with increased risk of CLABSIs with an odds ratio of 4.53, 4.54, 2.91, 4.56, 4.76, 3.74, 2.49, 2.41, 7.22, 6.77 and 5.16 respectively (p<0.05). Further, factors such as older age (>12 months) and low PRISM III score (≤ 15) significantly reduce the risk of CLABSIs by 83.64% and 69.14% respectively (p<0.05).

Conclusion

In conclusion, our results revealed that factors such as young age, high PRISM III score, leukocytosis, neutrophilia, anemia, change of central venous catheter, duration of catheterization (> 7 days), exposure to blood products during the hospital stay, use of steroids, inotropes, and prophylactic antibiotics were identified as risk factors for CLABSI.

## Introduction

Central line-associated bloodstream infection (CLABSI) is the most common hospital-acquired infection (HAI) accounting for 20-30% of all HAIs, followed by ventilator-associated pneumonia (20-25%), catheter-associated urinary tract infection (15%), surgical site infection (11%) and others [1]. In developing countries like India, the incidence of CLABSI varies from over 15 per 1000 catheter days and accounts for around 12-15% risk of mortality [1-12]. Recent studies reported that factors such as young age, gender, high pediatric risk of mortality (PRISM III) score, malnutrition, total parenteral nutrition, number of relevant hematological factors, catheter-related factors such as the site of catheter insertion, duration of central venous catheterization, use of medications such as steroids and prophylactic antibiotics increases the risk of HAIs such as CLABSI [1-12]. Studies also reported that the majority of CLABSIs were caused by gram-negative organisms with Klebsiella pneumonia being the most common etiology [11-15]. However, studies evaluating the factors associated with CLABSI and its etiology in India were limited. Our study aimed to evaluate the factors associated with the risk of CLABSI such as age, gender, PRISM III score, relevant hematological factors, catheter-related factors, medications, and profile of organisms causing CLABSI.

## Materials and methods

This is a prospective, single-center study conducted for a period of one year (January 2021 to February 2022) in the pediatric intensive care unit (PICU) of our hospital. The PICU receives patients from the emergency department, outpatient department, referrals from other hospitals, and postoperative patients requiring PICU care. The study was approved by our institutional ethical review board (Reference ID: ECASM-AIMS-2021-346). A minimum sample size of 54 was calculated with an 80% power and a 95% confidence interval based on the previous study [9]. Our study included 118 patients aged two months to 15 years admitted to our PICU in whom a central venous catheter was inserted for more than 48 hours. Patients with any previous infections and central venous catheter lines inserted outside our PICU were excluded. Patients’ demographics, relevant clinical parameters, central venous catheter-related factors, pediatric risk of mortality (PRISM III) scores, bacterial etiology, and outcomes were collected in a predesigned standardized form. A CLABSI was defined as a primary bloodstream infection in a patient who had 48 hours of central venous catheterization and is not related to infection at other sites [1, 10, 11, 13]. The PRISM III score was scored within 24 hours of hospital admission. Blood samples collected for blood culture were inoculated into brain heart infusion broth and incubated at 37°C for 24 hours in our hospital microbiological laboratory. Then subcultures were performed on blood agar (BA) along with gram films and antimicrobial susceptibility testing was performed by BACTEC (Becton Dickinson Diagnostic Systems, Franklin Lakes, NJ) [11, 16]. Blood culture was considered negative if no growth was identified on the final subculture after seven days. Blood culture was considered positive if any growths were identified based on morphology and biochemical reactions during the seven days. Patients were managed as per institutional PICU protocols and antibiotics were administered based on antimicrobial susceptibility.

Statistical analysis

Statistical analysis was performed using R statistical software for Windows, v. 4.1.0 (R Foundation for Statistical Computing, Vienna, Austria) [17-19]. Continuous variables were depicted as mean ± standard deviation (SD) and categorical variables were depicted as frequency and percentages. Pearson's chi-squared test with Yates' continuity correction and logistic regression with odds ratio were calculated by the “epitools” package [19]. A p-value of less than 0.05 was considered statistically significant.

## Results

Out of the 518 patients admitted to the PICU, our study included 118 patients with central venous catheterization (CVC). Among the 118 patients with CVC included in our study, 27% (32) of patients had CLABSI with an observed incidence of 61.78 CLABSIs per 1000 PICU admissions and 30.42 CLABSIs per 1000 central venous catheter days. Table 1 shows the characteristics of patients included in our study such as age, gender, diagnostic categorization, nutritional status, PRISM III score, hematological factors, catheter-related factors, exposures to medications, and outcomes. Patients with CLABSI under the age of 12 months were significantly higher than the patients without CLABSI (p = 0.0011). The mean (± SD) age of patients included in our study was 57.75 ± 40.28 months in the CLABSI groups and 60.15 ± 31.90 months in the non-CLABSI groups. Among the patients with CLABSI, no statistically significant difference was observed in gender (p = 0.3374) as shown in Table 1. Higher PRISM III scores of >15 (21.87%) were significantly associated with patients with CLABSI as compared to patients without CLABSI (p = 0.0262) as shown in Table 1. Four (12.5%) patients with CLABSI died during their PICU stay. Further, we observed a significant association between CLABSI and factors such as leukocytosis, neutrophilia, anemia, use of steroids, inotropes, and prophylactic antibiotics as shown in Table 1. However, we observed no significant association between CLABSI and factors such as nutritional status, diagnostic categorization (medical and surgical), other hematological factors (such as lymphocytosis and thrombocytosis), catheter-related factors, exposure to blood products, total parenteral nutrition and overall mortality in the analysis of Pearson's chi-squared test with Yates' continuity correction as statistical significance.

**Table 1:** Characteristics of patients included in our study. CLABSI: Central line-associated bloodstream infections. PRISM: Pediatrics risk of mortality score. CVC: Central venous catheter. The p-value was calculated by Pearson's Chi-squared test with Yates' continuity correction and a p-value < 0.05 was considered statistically significant.

Characteristics	CLABSI, n (%) Total = 32	Non – CLABSI, n (%) Total = 86	p-value
Age			
2-12 months	23 (71.87)	31 (36.05)	0.0011
> 12 months	9 (28.13)	55 (63.95)	
Gender			
Female	18 (56.25)	38 (44.19)	0.3374
Male	14 (43.75)	48 (55.81)	
Diagnosis categorization			
Surgical	6 (18.75)	24 (27.91)	0.4367
Medical	26 (81.25)	62 (72.09)	
Nutritional status			
Malnutrition	17 (53.13)	41 (47.67)	0.7494
PRISM III score			
≤ 15	25 (78.13)	81 (94.19)	0.0262
> 15	7 (21.87)	5 (5.81)	
Hematological factors			
Leucocytosis	22 (68.75)	37 (43.02)	0.0227
Neutrophilia	22 (68.75)	28 (32.55)	0.0009
Lymphocytosis	7 (21.86)	16 (18.60)	0.8908
Anemia	26 (81.25)	41 (47.67)	0.0022
Thrombocytosis	8 (25.00)	12 (13.95)	0.2518
Catheter-related factors			
Change of CVC	6 (18.75)	5 (5.81)	0.0730
CVC days >7 days	24 (75.00)	47 (54.65)	0.0725
Exposures			
Blood products	13 (40.63)	19 (22.09)	0.0750
Total parenteral nutrition	5 (15.63)	5 (5.81)	0.1837
Steroids	18 (56.25)	13 (15.12)	0.00002
Inotropic agents	12 (37.5)	7 (8.14)	0.0003
Prophylactic antibiotics	20 (62.50)	21 (24.42)	0.0003
Outcome			
Recovery	28 (87.50)	69 (80.23)	0.5177
Dead	4 (12.50)	17 (19.77)	

Table 2 shows the association of factors such as age, PRISM III score, hematological factors (leukocytosis, neutrophilia, anemia), catheter-related factors (change of central venous catheter, duration of central venous catheterization), exposure to blood products and medications (use of steroids, inotropes, and prophylactic antibiotics) with CLABSI. Odds ratios with a 95% confidence interval were calculated by logistic regression analysis using R language and the methods were shown in the appendix section.

Statistical analysis showed that patients with central venous catheters in the age group of 2-12 months were significantly associated with increased risk of CLABSI by 4.53 fold (OR, 4.5340; 95% CI, 1.9199 to 11.4969; p = 0.0008) and patients in the age group of greater than 12 months were significantly associated with reduced risk of CLABSI by 83.64% (OR, 0.1636; 95% CI, 0.0755 to 0.3144; p = 0.0000005) in comparison to patients without CLABSI. Regarding the PRISM III score, a score of greater than 15 was significantly associated with increased risk of CLABSI by 4.54 fold (OR, 4.5360; 95% CI, 1.3348 to 16.5412; p = 0.0162) when compared to patients without CLABSI. Hematological factors such as leukocytosis, neutrophilia, and anemia were significantly associated with increased risk of CLABSI by 2.91 fold (OR, 2.9135; 95% CI, 1.2584 to 7.1331; p = 0.0149), 4.56 fold (OR, 4.5571; 95% CI, 1.9476 to 11.3054; p = 0.0007) and 4.76 fold (OR, 4.7561; 95% CI, 1.8788 to 13.8236; p = 0.0019) respectively when compared to patients without CLABSI. Catheter-related factors such as change of central venous catheter and duration of central venous catheterization for more than seven days were significantly associated with increased risk of CLABSI by 3.74 fold (OR, 3.7385; 95% CI, 1.0452 to 13.9567; p = 0.0413) and 2.49 fold (OR, 2.4894; 95% CI, 1.0396 to 6.4840; p = 0.0484) respectively. We also observed that exposure to blood products during the hospital stay was significantly associated with increased odds of CLABSI by 2.41 fold (OR, 2.4127; 95% CI, 1.0029 to 5.7783; p = 0.0473). Further, medications such as steroids, inotropes, and prophylactic antibiotics were significantly associated with increased odds of CLABSI by 7.22 fold (OR, 7.2198; 95% CI, 2.9456 to 18.5220; p = 0.00002), 6.77 fold (OR, 6.7714; 95% CI, 2.4171 to 20.3758; p = 0.0004) and 5.16 fold (OR, 5.1587; 95% CI, 2.2003 to 12.6207; p = 0.0002) respectively. When adjusted for PRISM III scores, our analysis showed that the use of steroids (OR = 6.60, p = 0.00007), inotropes (OR = 5.79, p = 0.00146), prophylactic antibiotics (OR = 5.41, p = 0.000247) were significantly associated with increased risk of CLABSI. However, exposure to blood products (OR = 2.40, p = 0.0552) was not significantly associated with CLABSI when adjusted for PRISM III scores.

**Table 2:** Factors associated with CLABSI. p < 0.05 was considered statistically significant. *p-value =  Pr(>|z|). CLABSI: central line-associated bloodstream infections. PRISM: pediatrics risk of mortality score. CVC: central venous catheter. CI: confidence interval

	CLABSI, n (%) Total = 32	Non-CLABSI, n (%) Total = 86	Coefficient estimate	Standard error	z-value	p-value*	Odds ratio	95% CI
Age								
2 - 12 months	23 (71.87)	31 (36.05)	1.5116	0.4528	3.338	0.0008	4.5340	1.9199 - 11.4969
> 12 months	9 (28.13)	55 (63.95)	-1.8101	0.3596	-5.034	0.0000005	0.1636	0.0755 - 0.3144
PRISM III score								
≤ 15	25 (78.13)	81 (94.19)	-1.1756	0.2288	-5.138	0.0000003	0.3086	0.1932 - 0.4757
> 15	7 (21.87)	5 (5.81)	1.5120	0.6287	2.405	0.0162	4.5360	1.3348 - 16.5412
Hematological factors								
Leucocytosis	22 (68.75)	37 (43.02)	1.0694	0.4392	2.435	0.0149	2.9135	1.2584 - 7.1331
Neutrophilia	22 (68.75)	28 (32.55)	1.5167	0.4454	3.405	0.0007	4.5571	1.9476 - 11.3054
Anemia	26 (81.25)	41 (47.67)	1.5594	0.5017	3.108	0.0019	4.7561	1.8788 - 13.8236
Catheter related factors								
Change of central venous catheter	6 (18.75)	5 (5.81)	1.3187	0.6461	2.041	0.0413	3.7385	1.0452 - 13.9567
Duration of central venous catheter > 7 days	24 (75.00)	47 (54.65)	0.9120	0.4622	1.973	0.0484	2.4894	1.0396 - 6.4840
Exposures								
Blood products	13 (40.63)	19 (22.09)	0.8808	0.4440	1.984	0.0473	2.4127	1.0029 - 5.7783
Steroids	18 (56.25)	13 (15.12)	1.9768	0.4665	4.238	0.00002	7.2198	2.9456 - 18.5220
Inotropes	12 (37.5)	7 (8.14)	1.9127	0.5374	3.559	0.0004	6.7714	2.4171 - 20.3758
Prophylactic antibiotics	20 (62.50)	21 (24.42)	1.6407	0.4431	3.703	0.0002	5.1587	2.2003 - 12.6207

Table 3 shows the microbial etiology in patients with CLABSI. We observed that 34.3% (11) of the samples tested were identified as gram-negative organisms, 21.8% (7) were identified as gram-positive organisms, 6.3% (2) were due to Candida albicans and 37.5% (12) were identified as polymicrobial growth based on blood culture. Of the five Klebsiella pneumonia isolates, three were multidrug-resistant and two were sensitive to carbapenem and extended-spectrum beta-lactamase (ESBL). Out of the four Acinetobacter baumani isolates, two were multidrug-resistant and two were sensitive to carbapenem and ESBL. Both Burkholderia cepacia and Salmonella were sensitive to carbapenem and ESBL. Among the four coagulase-negative staphylococci isolates, three were methicillin-resistant and one was methicillin-sensitive, whereas both isolates of Staphylococcus aureus were methicillin-resistant. Among the Candida albicans, one was fluconazole-resistant and one was fluconazole sensitive. 

**Table 3:** Microbial etiology in patients with CLABSI.

Etiology of organism	CLABSI, n (%) Total = 32
Klebsiella pneumonia	5 (15.6%)
Acinetobacter baumani	4 (12.5%)
Coagulase-negative staphylococci	4 (12.5%)
Candida albicans	2 (6.3%)
Staphylococcus aureus	2 (6.3%)
Burkholderia cepacia	1 (3.1%)
Salmonella	1 (3.1%)
Diphtheroid	1 (3.1%)
Polymicrobial	12 (37.5%)

## Discussion

Our study included 118 patients with central venous catheterization in the age group of two months to 15 years. Out of the 118 patients included in our study, a high proportion of 61.78 CLABSIs per 1000 PICU admissions and 30.42 CLABSIs per 1000 central venous catheter days were observed as compared to the incidence range of 3.9 to 17.04 per 1000 central venous catheter days reported in the previous studies [8, 10-14]. The observed 30.42 CABSIs per 1000 central venous catheter days in our study were lower than the 47.31 bloodstream infections associated with central venous catheterization per 1000 catheter days previously reported in the adult population [20]. We also observed a female predominance of CLABSI cases with no statistical significance in contrast to the results reported in previous studies [3, 4, 9, 10, 20]. We observed an overall mortality of 12.5% in patients with CLABSI compared to the mortality of 12.9% to 56% reported in patients with CLABSI in previous studies [10-13]. This may be attributed to the lack of analyses based on the severity of the disease. Among the four deaths in patients with CLABSI, two had a PRISM III score of > 15 and another two had a PRISM III score of ≤ 15, whereas among the 17 deaths in patients without CLABSI, three had a PRISM III score of > 15 and 14 had PRISM III score of ≤ 15. Our analysis showed that factors such as patients in the age group of 2-12 months, high PRISM III score of greater than 15, leukocytosis, neutrophilia, anemia, change of central venous catheter, duration of central venous catheterization for more than seven days, exposure to blood products during the hospital stay, use of steroids, inotropes and prophylactic antibiotics were significantly associated with increased risk of central line-associated bloodstream infections (CLABSIs) as shown in Table 2. The reported factors such as age, change of central venous catheter, and duration of catheterization were consistent with the previous reports [10-13]. In our study, the central venous lines in 11 patients were changed after seven days of catheterization; this was found to be significantly associated with CLABSI. However, further studies addressing the change of central venous catheters before and after seven days were needed to validate these findings. Further, a significant association of higher PRISM III scores indicates an association with the increased severity of the disease. Exposure to blood products during the hospital stay, exposure to steroids, and exposure to previous antibiotics were found to be significantly associated with the risk of CLABSI and our results were consistent with the previous reports [12]. However, the significant association between CLABSI and the exposure to vasoactive agents (inotropic agents) reported in our study was contrary to the previous reports [12]. Moreover, factors such as the use of steroids, inotropes, and prophylactic antibiotics were significantly associated with CLABSI when adjusted for the PRISM III scores (severity of disease). However, exposure to blood products was not significantly associated with CLABSI when adjusted for PRISM III scores. Further, factors such as patients in the age group of greater than 12 months and patients with low PRISM III score of ≤ 15 were significantly associated with reduced risk of central line-associated bloodstream infections (CLABSIs) by 88.44% and 69.14% respectively as shown in Table 2. The observed major site of insertion was the internal jugular vein in our study which is consistent with the previous studies [21, 22] and contradictory to those reported by Tomar et al [11].

Overall, we observed that Klebsiella pneumonia was the most common organism causing CLABSI in our study which is consistent with those results previously reported in India [10-13, 15, 20]. Further, we observed that the majority of CLABSIs were caused by gram-negative organisms in our study which are consistent with those reported in the previous studies [10-15, 20]. However, some studies reported that gram-positive organisms were the most common etiology of CLABSI, which is contradictory to the results reported in our study [1, 23, 24]. Regarding antimicrobial susceptibility, we observed that overall 15.62% were multidrug-resistant bacteria with 18.75% susceptible to carbapenem and extended-spectrum beta-lactamase which is consistent with rates reported in previous studies [11, 13]. We also observed that 50% of Staphylococcus aureus and 75% of coagulase-negative staphylococcus were methicillin-resistant and 50% of candida species were fluconazole-resistant, which is consistent with those reported in the previous studies [10, 13].

Overall, the results observed in our study underscore the need for continuous surveillance and robust multidisciplinary prevention strategies such as providing bundle care approaches and antibiotic stewardship programs [25, 26]. The bundle of care approach is an evolving multidisciplinary approach that focuses on prevention strategies such as primary prevention (neonate, staff, caretaker, environment, devices), secondary prevention (detection, screening, and epidemiological surveillance), tertiary prevention (antibiotic prescription, stewardships, outbreak controls) and implementation of the bundle care practices (by audit, feedback, awareness, organizing implementation team meetings, education, training, licensure standards) to promote and sustain the implementation of these practices in the hospitals [25, 26]. Further, providing education to the health care workers regarding the antibiotic stewardship practices such as promoting appropriate selection of treatment regimens and prevention of unnecessary exposure to antibiotics might reduce the emergence of antibiotic resistance [27]. We also suggest that close adherence to the bundle of care practices regarding central venous catheter insertion and maintenance and education to healthcare workers about the maintenance of catheters might help in tackling the increased prevalence of CLABSIs [25, 26]. Further, we also suggest that antibiotic optimization policy and guidance protocols based on regional data on antimicrobial susceptibility need to be regularly updated to combat the increased incidence of antimicrobial resistance [25-27].

Moreover, there are some limitations to be considered when interpreting our results such as small sample size, short duration of study, and lack of 28-day mortality rates and data regarding the care provided during central venous catheter insertion, access, and maintenance. However, our study provided a prospective and comprehensive analysis of factors associated with CLABSI and the profile of organisms in the pediatric intensive care unit in southern India when compared to previous studies. Further, prospective studies with large sample sizes and longer duration, and involving multiple centers are needed to validate these findings and generalize these results for clinical implementation.

## Conclusions

In conclusion, our results revealed that factors such as patients in young age, high PRISM III scores, leukocytosis, neutrophilia, anemia, change of central venous catheter, duration of central venous catheterization for more than seven days, use of steroids, inotropes, and prophylactic antibiotics significantly increases the risk of CLABSI. However, factors such as being older than 12 months of age and having lower PRISM III scores play a protective role in significantly reducing the risk of CLABSIs. Moreover, long-term multicentric and interventional studies with large sample sizes were needed to confirm our findings. Further, robust analysis with additional factors based on the severity of the diseases was needed.

## Appendices

The methods used for the statistical analysis in R language using R-statistical software for windows version 4.1.0 is shown in this appendix section.

# Packages used were ‘readxl’ and ‘epitools’

# To calculate Pearson's Chi-squared test with Yates' continuity correction for each factors.

>  factortable <- table(data$clabsi, data$factors)

> chisq.test(factortable)

# output for Chi-squared test.

# To perform logistic regression analysis and odds ratio with 95% confidence intervals

> logitmodel <-glm(clabsi~factors, family = 'binomial', data = data)

> summary(logitmodel)

# output for logistic regression analysis.

> exp(cbind(ODDS=coef(logitmodel), confint(logitmodel)))

# output for odds ratio with 95% confidence interval.
